# DNA methylation alterations caused by *Leishmania* infection may generate a microenvironment prone to tumour development

**DOI:** 10.3389/fcimb.2022.984134

**Published:** 2022-08-29

**Authors:** Ana Florencia Vega-Benedetti, Eleonora Loi, Patrizia Zavattari

**Affiliations:** Department of Biomedical Sciences, Unit of Biology and Genetics, University of Cagliari, Cagliari, Italy

**Keywords:** DNA methylation alterations, CpG sites, *Leishmania* infection, chronic infection, cancer onset, pathogen-associated cancer, *Leishmania*/host cell interaction

## Abstract

DNA methylation is an epigenetic signature consisting of a methyl group at the 5’ cytosine of CpG dinucleotides. Modifications in DNA methylation pattern have been detected in cancer and infectious diseases and may be associated with gene expression changes. In cancer development DNA methylation aberrations are early events whereas in infectious diseases these epigenetic changes may be due to host/pathogen interaction. In particular, in leishmaniasis, a parasitic disease caused by the protozoan *Leishmania*, DNA methylation alterations have been detected in macrophages upon infection with *Leishmania donovani* and in skin lesions from patients with cutaneous leishmaniasis. Interestingly, different types of cancers, such as cutaneous malignant lesions, lymphoma and hepatocellular carcinoma, have been diagnosed in patients with a history of leishmaniasis. In fact, it is known that there exists an association between cancer and infectious diseases. *Leishmania* infection may increase susceptibility to develop cancer, but the mechanisms involved are not entirely clear. Considering these aspects, in this review we discuss the hypothesis that DNA methylation alterations induced by *Leishmania* may trigger tumorigenesis in long term infection since these epigenetic modifications may enhance and accumulate during chronic leishmaniasis.

## Graphical Abstract

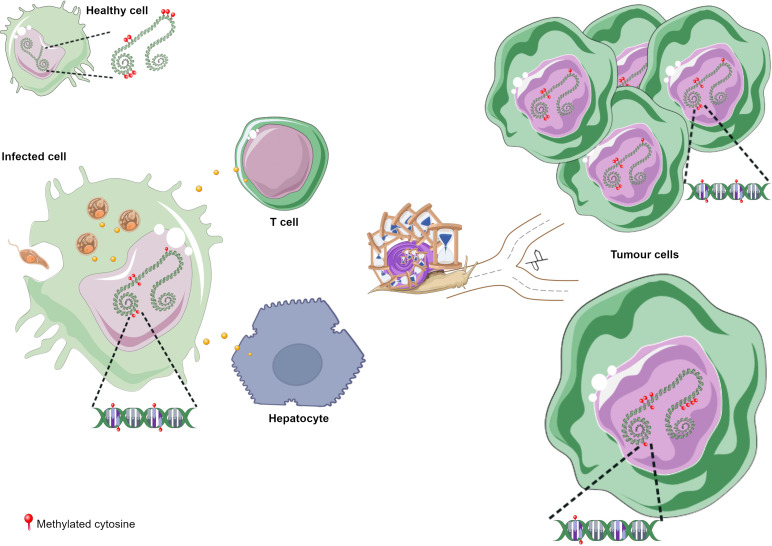

## Introduction

Several works reported a possible association between infectious diseases and cancer development but the mechanisms leading to malignant transformation are not well elucidated (Kopterides et al., 2007; Al-Kamel, 2017; van Tong et al., 2017; Yasunaga and Matsuoka, 2018). Epigenetics is an interesting research area whose study is increasing in pathogen diseases while it is widely investigated in cancer. It refers to heritable and reversible changes that affects the genetic material packaging and expression without altering the DNA sequence. The epigenetic regulatory network mainly includes DNA methylation, histone modifications and non-coding RNAs (Gal-Yam et al., 2008; Saavedra et al., 2012). Pathogens hijack the epigenome of host cells generating a suitable environment for their survival and replication. DNA methylation, an epigenetic sign, is modulated by pathogens during their interaction with host cells. The DNA methylation profile is relevant for gene expression regulation and chromosome stability. Bacteria, virus and parasites may alter the host methylome and the enzymes or cofactors involved in DNA methylation modulation (Silmon de Monerri and Kim, 2014). In particular, several DNA methylation aberrations have been detected in macrophages upon infection with the protozoan *Leishmania* spp., responsible for leishmaniasis, a neglected tropical disease (Marr et al., 2014).

Alterations in DNA methylation pattern are also early events in cancerogenesis, and they are tumour-specific signs. Therefore, they are considered excellent diagnostic and useful prognostic biomarkers (Gal-Yam et al., 2008). These aberrations frequently lead to downregulation of tumour suppressor genes and upregulation of oncogenes (Skvortsova et al., 2019). Pathogen infections such as leishmaniasis can be considered tumour promoters since they generate an environment prone to malignancy development employing host colonization strategies similar to cancer cells. In fact, cancer cases have been reported in patients with a history of *Leishmania* infection (Kopterides et al., 2007; Schwing et al., 2019). The novelty of the present review resides in the description of a possible association between *Leishmania* infection and cancer onset due to changes in the DNA methylation profile.

## DNA methylation

DNA methylation is an epigenetic signature consisting of a methyl group at the 5’ cytosine of CpG dinucleotides. CpG sites are distributed across the genome, in regulatory regions, at DNA repetitive elements and gene bodies. Regions with high CpG content termed CpG islands (CGI) are often located at gene promoter regions. DNA methylation maintains genome stability and regulates gene expression, in particular when methylated CpG sites are located in promoter regions, *i.e.* a high content of methylated cytosines is generally associated with transcriptional downregulation whereas a hypomethylated state is frequently linked to gene expression upregulation. The addition of the methyl group can be maintained during cell division but it is also a reversible modification (Gal-Yam et al., 2008; Ambrosi et al., 2017). Two protein families, DNA methyltransferases (DNMTs) and Ten-eleven translocation (TET), together with protein partners, are relevant players of the DNA methylation pathway. DNMTs include three enzymes; DNMT1 restores the methylation pattern of the new DNA strand during replication, whereas DNMT3A and DNMT3B with their cofactor DNMT3L are involved in *de novo* DNA methylation. DNMT1 and DNMT3 mainly differ at the N-terminal domain mediating their specific activity at genomic sites. DNMTs interact with several accessory molecules such as DNMT3L, UHRF1 and RNAs. DNMT3L acts during genomic imprinting establishment regulating DNMT3-target specificity and enzyme activity, while UHRF1 recruits DNMT1 to hemimethylated DNA during replication (Lee et al., 2014; Ambrosi et al., 2017). On the other hand, DNA demethylation is catalysed by TET enzymes which are responsible for 5-methylcytosine oxidation whose products are removed by the DNA repair mechanism. TET family consists of three members which contain or interact with CXXC domains, key sequences in CpG regions recognition (Schubeler, 2015; Ambrosi et al., 2017). The expression of DNMT and TET enzymes varies during development leading to DNA methylation pattern changes that, with other epigenetic mechanisms, participate in the silencing or activation of pathways for cellular stemness, differentiation, proliferation, among others. For instance, TET1 and TET2 maintain stemness phenotype by interaction with NANOG, a pluripotency protein (Lee et al., 2014; Ambrosi et al., 2017).

### DNA methylation alterations in cancer and infectious diseases

**Figure 1:**
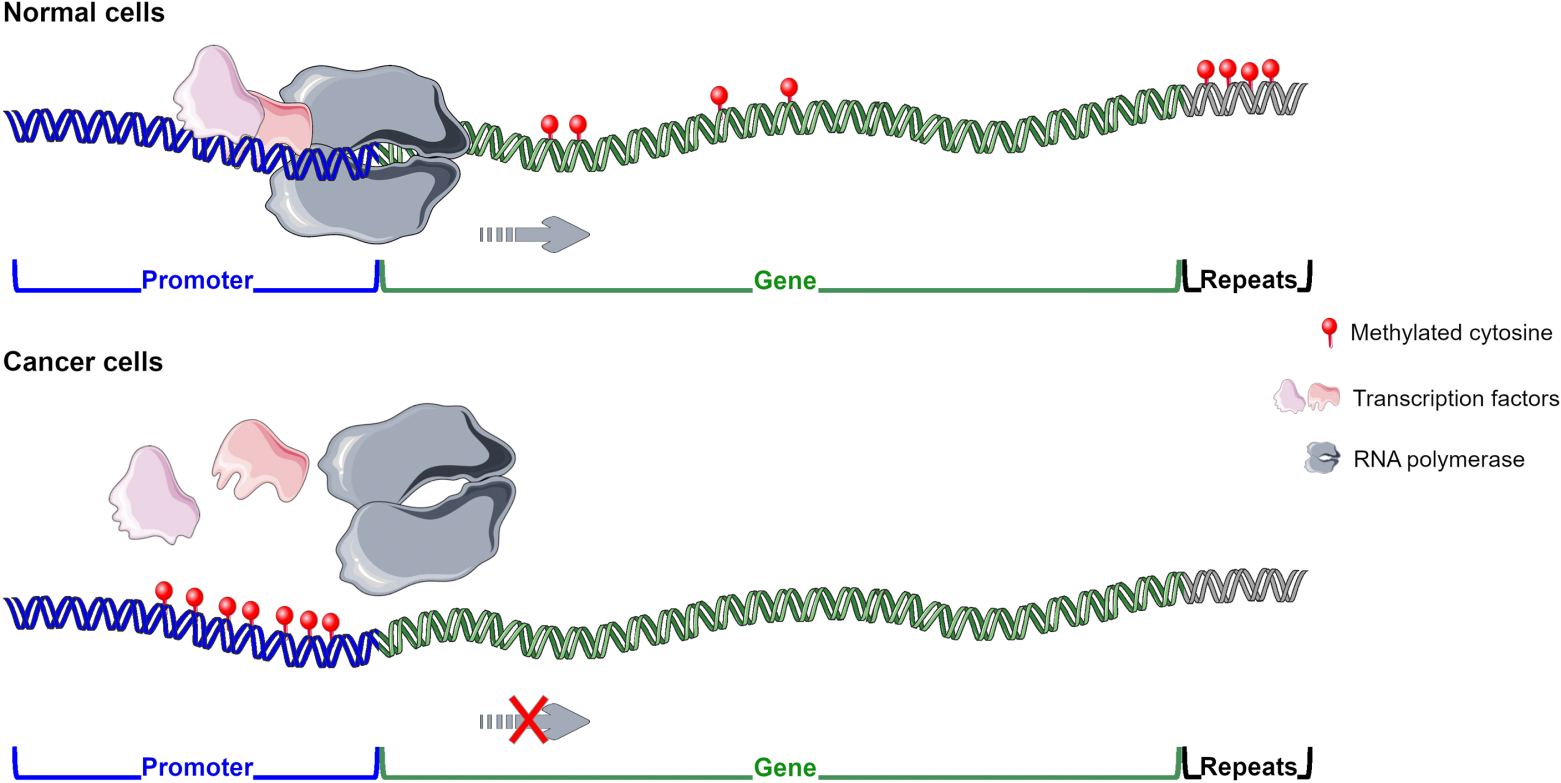
DNA methylation pattern in normal and cancer cells. In normal cells methyl group is widely distributed except for CGIs located at promoters and enhancers of tumour suppressor genes enabling their transcription. In cancer cells hypermethylation is generally observed at regulatory regions of tumour suppressor genes inhibiting their expression, whereas global hypomethylation is detected. Figure created using Servier Medical Art images, licensed under a Creative Commons Attribution 3.0 Unported License; https://smart.servier.com/.

DNA methylation alterations have been reported in different pathologies, including cancer and infectious diseases. Normal cells present global hypermethylation and unmethylated CGIs at promoter regions, whereas cancer cells undergo a global hypomethylation and hypermethylation at the 5’ gene regulatory regions (Gal-Yam et al., 2008). High content of methylated CpGs at promoter regions, frequently belonging to tumour suppressor genes, is generally associated with gene expression downregulation (Fadda et al., 2018; Loi et al., 2019; Skvortsova et al., 2019; Vega-Benedetti et al., 2020; Vega-Benedetti et al., 2022) (**Figure 1**). Methylated cytosines can affect transcription factor (TF) binding and thus the recruitment of the RNA polymerase to the transcription start site. For example, Sp1 cannot bind to its consensus site at the Retinoblastoma (*RB*) gene promoter whether it contains methylated CpGs (Clark et al., 1997; Heberle and Bardet, 2019). Gain of methylation at enhancer regions could also disrupt normal TF binding contributing to tumour suppressor gene downregulation (Schubeler, 2015; Skvortsova et al., 2019) (**Figure 1**). DNA methylation differently influences TF binding and thus its function (Yin et al., 2017). Instead, in several cancers, such as Wilms tumour, ovarian and breast carcinomas, hypomethylation in satellite DNA could predispose to chromosomal translocations (Feinberg and Tycko, 2004; Gal-Yam et al., 2008). Loss of methylation at retrotranspons may reactivate them leading to possible dysregulation of normal gene expression (Suter et al., 2004). Demethylation in cancer can also occur at promoter regions of oncogenes increasing or reactivating their transcription. DNA methylation works in combination with histone modifications and nucleosome organization to regulate gene expression (Skvortsova et al., 2019). Several experimental studies have been performed to demonstrate the role of DNA methylation changes in tumorigenesis. For instance, in mice models induced hypomethylation led to chromosome instability and tumour promotion, whereas DNMT inhibition prevented malignancy development (Gal-Yam et al., 2008; Kulis and Esteller, 2010). DNA methylation alterations are cancer-specific early events in tumorigenesis resulting in promising diagnostic biomarkers. These aberrations have been found in preneoplastic and histologically normal tissues of people susceptible to develop cancer (Feinberg and Tycko, 2004; Gal-Yam et al., 2008; Saavedra et al., 2012; Luo et al., 2014; Wong Doo et al., 2016; Micevic et al., 2017; Fadda et al., 2018; Loi et al., 2019). To note, methylated-based biomarkers can be traced in cell-free DNA from several matrices including plasma, stool, urine and bile (Gal-Yam et al., 2008; Fadda et al., 2018; Vega-Benedetti et al., 2020; Loi et al., 2022). Moreover, DNA methylation alterations are useful prognostic biomarkers in several cancers including cutaneous melanoma, lung adenocarcinoma, ovarian, breast and cervical cancer (Fleischer et al., 2014; Guo et al., 2018; Guo et al., 2019; Wang et al., 2019; Yang et al., 2020).

On the other hand, in infectious diseases microbes hijack the epigenome of host cells, including DNA methylation, to elude host defensive mechanisms and promote their survival. Previous evidence showed that virus, bacteria and parasites manipulate the transcription of host defence genes leading to immunosuppression (Paschos and Allday, 2010; Gómez-Díaz et al., 2012; Silmon de Monerri and Kim, 2014). To achieve this aim different strategies are employed, for instance viral DNA integration in host genome induces DNA methylation changes in flanking regions, enabling viral latency. Modulation of host enzymes, such as the DNA methyltransferases, promotes modifications in DNA methylation pattern during hepatitis B (HBV) and Epstein-Barr virus (EBV) infections (Silmon de Monerri and Kim, 2014). *Helicobacter pylori* infection induces epigenome modification of host cells such as hypermethylation of *FOXD3* promoter, a key participant in apoptosis. The bacteria *Mycobacterium leprae* facilitates its dissemination in the host through DNA demethylation at promoters of genes involved in the epithelial-mesenchymal transition (Silmon de Monerri and Kim, 2014). Regarding parasites, they may regulate host DNA methylation pattern during cell invasion or through protein secretion delivered by vesicles (Silmon de Monerri and Kim, 2014). *In vivo* and *in vitro* assays showed DNA methylation modifications upon *Toxoplasma gondii* and *Leishmania donovani* infection (Hari Dass and Vyas, 2014; Marr et al., 2014). In agreement with pathogen modulation of host defence system, many altered genes upon *Leishmania* infection participate in host immune response including the following mechanisms: cytotoxicity mediated by Natural killer cells, interaction between cytokines and their receptors, chemokine/adipocytokine signalling and leukocyte migration (Marr et al., 2014).

## *Leishmania* infection: Host/parasite interplay

Leishmaniasis is a neglected tropical disease caused by different species of the protozoan *Leishmania* such as *L. braziliensis*/*amazonensis*, responsible for the development of the cutaneous and muco-cutaneous clinical forms, and *L. infantum*/*donovani*, causative agents of visceral leishmaniasis (Afrin et al., 2019; WHO, 2020). This parasite has a complex life cycle, alternating two stages: the flagellated promastigote and the amastigote forms. Once it is injected in the host by the bite of a female sandfly (*Lutzomyia* species), promastigotes enter macrophages where they replicate as amastigotes. *Leishmania* parasites successfully colonize macrophages due to their capability to avoid host defence mechanisms by modulating their surface molecules and host immune response including macrophage activation and antigen processing (Robert McMaster et al., 2016; Afrin et al., 2019). These strategies favour protozoan survival and establishment within the host (Marr et al., 2014; Robert McMaster et al., 2016; Al-Kamel, 2017). Interestingly, parasites manipulate host epigenome. *i.e.* DNA methylation, histone modifications and non-coding RNA, altering gene expression and thus signalling pathways (Parmar et al., 2020; Roy et al., 2020). Marr et al. reported several differentially methylated CpG sites in macrophages upon infection with *L. donovani* (Marr et al., 2014). Genes affected by these altered features are implicated in the following signalling pathways: JAK/STAT, MAPK, Notch and Wnt signalling, focal adhesion, among others (Marr et al., 2014). Moreover, low methylation level at *FLI1* promoter was observed in cutaneous lesions from patients with *L. braziliensis* infection and in IL-6 treated macrophages infected with the same *Leishmania* sp. (Almeida et al., 2017). As observed in the experiments performed by Almeida et al., IL-6 affects methylation of CpGs in the *FLI1* promoter suggesting that cellular communication and the surrounding environment are important variables during parasite/host interaction (Almeida et al., 2017).

Although macrophages are the main host cells for *Leishmania* spp., dendritic cells responsible for antigen presentation also phagocytose parasites and migrate to lymph nodes. *Leishmania* infection progression is mainly regulated by a complex interplay between macrophages and dendritic cells. IL-12 produced by infected dendritic cells triggers a cascade of events leading to macrophage classical activation involved in parasiticidal activity, whereas IL-10 and TGF-β released by infected macrophages result in alternative macrophage activation and thus in parasite survival (Liu and Uzonna, 2012). However, some *Leishmania* species such as *L. infantum* present high tropism to hepatocytes establishing a strong interaction with their membrane and regulating gene expression although parasite internalization has not been confirmed (Kausalya et al., 1993; Rodrigues et al., 2019). Little is known about the factors that guide *Leishmania* tissue tropism (Seblova et al., 2015). Few genes that may contribute to the disease tropism have been identified in different *Leishmania* species (Peacock et al., 2007). Tropism may also depend on host susceptibility including immunity and genetics, and on parasite virulence (Chang and McGwire, 2002; Reithinger et al., 2002).

This evidence suggests that *Leishmania* is able to hijack the epigenome, in particular DNA methylation, and regulate the transcriptional machinery not only of antigen-presenting cells but also of other cell types located at the infection site, such as fibroblasts and hepatocytes (Almeida et al., 2017; Rodrigues et al., 2019). It is not clear whether these changes occur due to molecules, including methyltransferase inhibitors, activators, and ncRNAs, released by *Leishmania* spp. and delivered *via* exosomes and/or microvesicles before parasite internalization and/or only when the pathogen is already inside macrophages (Silverman et al., 2010a; Coakley et al., 2015; Lambertz et al., 2015; Robert McMaster et al., 2016). Since parasite internalization is not reported in some immune cells, such as T cells, and in non-immune cells, extracellular vesicles communication may have a key role in cell epigenome modification. It has been reported that extracellular vesicles released by *L. major* target T cells leading to an increase production of IL-4 (Silverman et al., 2010b; Coakley et al., 2015). The interaction between the molecules released by parasites and the host epigenome is not fully established. Of note, the glycoprotein GP63 and the elongation factor-1α (EF-1α) secreted by *Leishmania* spp. have been reported to alter host pathways regulating kinases and phosphatases activity (Lambertz et al., 2012; Robert McMaster et al., 2016). Other released molecules, LmS3a, a ribosomal protein, and lipophosphoglycan glycoconjugates (LPP) regulate T-cell activation, iNOS gene expression and nitric oxide production (Ouaissi and Ouaissi, 2005). Parasites, including *Leishmania* spp. and *Trypanosoma cruzi*, also alter exosomes content and release from the infected cells. In this way, they are able to regulate the crosstalk between host cells and thus the host response against infection. This evidence supports vesicles as an useful signal transmission mechanism between parasites, parasites and host cells, and from host cells to the surrounding environment (Silverman et al., 2010a; Hassani and Olivier, 2013; Coakley et al., 2015). Therefore, in leishmaniasis exosomes may be an alternative system for molecules delivery to different cell types, such as T cells, fibroblasts and hepatocytes, possibly leading to their function dysregulation.

## Association between infectious diseases and cancer

**Figure 2:**
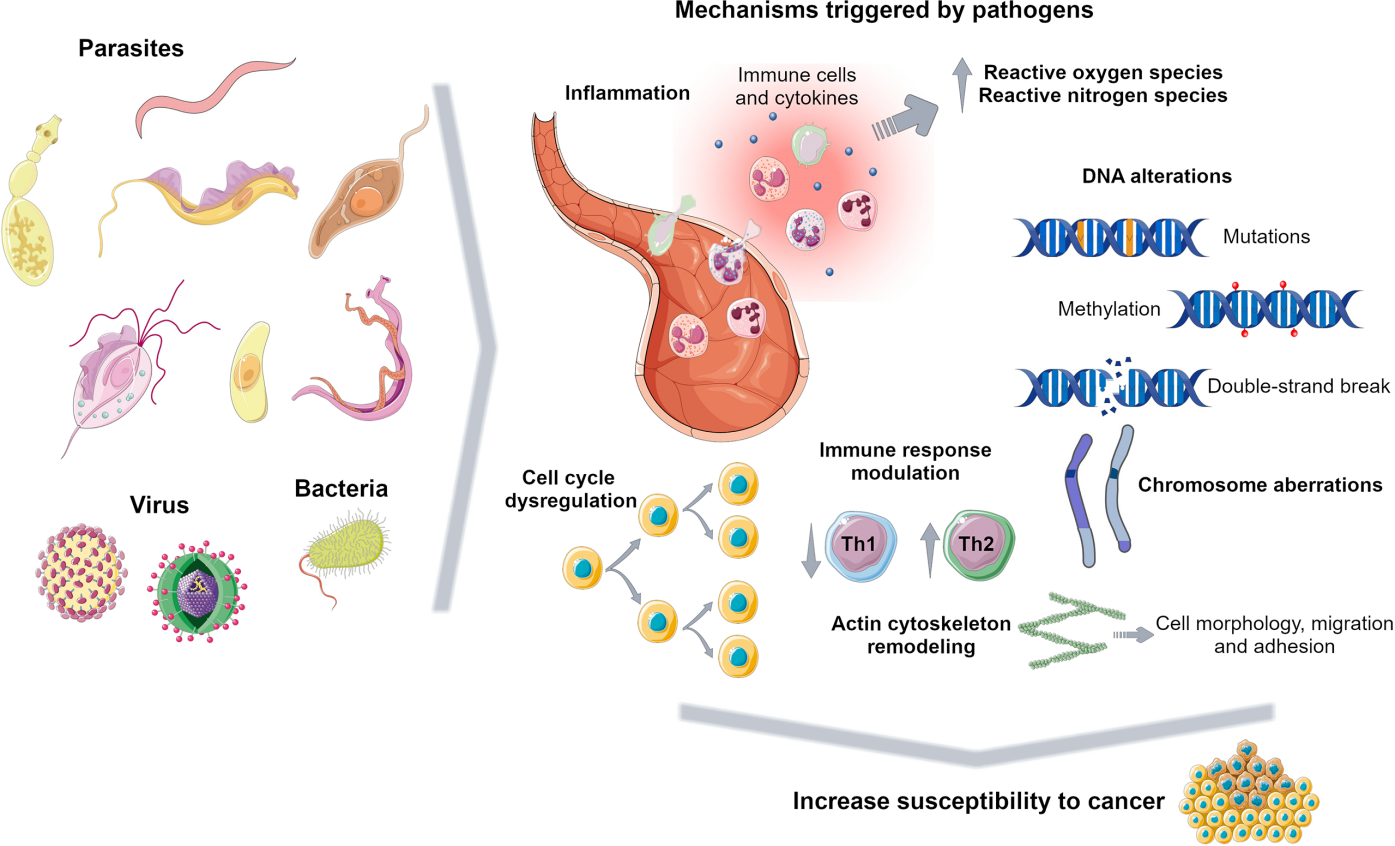
Carcinogenic mechanisms triggered by pathogens. Pathogens promote several events to survive in the host including inflammation, DNA alterations, immune response dysregulation and cell cycle modulation, increasing susceptibility to tumorigenesis. Figure created using Servier Medical Art images, licensed under a Creative Commons Attribution 3.0 Unported License; https://smart.servier.com/.

Previous works reported a casual association between cancer and infectious disease including bacteria, virus and parasite infection (Machicado and Marcos, 2016; van Tong et al., 2017; Yasunaga and Matsuoka, 2018; Schwing et al., 2019). Pathological conditions due to infectious diseases contribute to tumour onset and on the other hand cancer cells and surrounding environment lead to a susceptible landscape for pathogen infection (Schwing et al., 2019). Pathogens promote several carcinogenic mechanisms including chronic inflammation, immune response modulation and a series of other events that may trigger further cell function alterations (**Figure 2**).

### Infection-associated inflammation

Inflammation is a biological response to cellular damage due to injury or infection and its chronicity increases cancer risk (Shalapour and Karin, 2015). It enhances reactive oxygen and nitrogen species production leading to DNA damage (Machicado and Marcos, 2016; Schwing et al., 2019). For instance, it has been reported that liver fluke infection results in an overproduction of free radicals due to the inflammatory environment consisting of eosinophils, macrophages, neutrophils and the released cytokines. On one hand, a persistent oxidative stress condition triggers lipid peroxidation whose toxic products together with free radicals may induce DNA damage and dysregulate cell homeostasis (Andrade et al., 2012; Bahrami et al., 2014; Kim et al., 2016). On the other hand, *Clonorchis sinensis*-associated prolonged inflammation maintains elevated cytokines and NF-kB levels inducing further proinflammatory responses such as nitric oxide production, responsible for the DNA repair inhibition and the induction of *COX-2* expression, involved in cell growth modulation (Kim et al., 2016). This persistent environment promotes cholangiocarcinoma development (Kim et al., 2016). *Trichomonas vaginalis* infection increases susceptibility to develop prostate cancer through a secreted protein triggering cell proliferation and inflammation, possibly contributing to angiogenesis (Twu et al., 2014). In leishmaniasis chronic inflammation due to parasite persistence orchestrates a tumour microenvironment characterized by hypoxia, altered expression of *COX-2* and *NF-kB*-target genes (Gregory et al., 2008; Charpentier et al., 2016; Al-Kamel, 2017). However, *Leishmania* upregulates anti-inflammatory response reducing NF-Kb and inflammasome activation and increasing IL-10 to generate a safe niche for its survival (Mahanta et al., 2018; Afrin et al., 2019; Lecoeur et al., 2020; Parmar et al., 2020). *Leishmania* may disturb oxygen supply to the tissue lesions contributing to a complex scenario including proinflammatory and anti-inflammatory macrophages (Charpentier et al., 2016; Schatz et al., 2018; Saunders et al., 2021).

### Genomic instability induced by infections, a malignancy promoter

Genomic instability, a carcinogenic mechanism, including point mutations, structural chromosomal aberrations and DNA strand breaks, can be caused by pathogen infections (van Tong et al., 2017). In fact, an association between parasitic infections and lymphoid neoplasia has been reported. Burkitt’s lymphoma (BL) cases are common in endemic malaria regions with high *Plasmodium falciparum* transmission. *P. falciparum* may induce *C-Myc* translocation in B cells predisposing patients to develop BL (Torgbor et al., 2014; van Tong et al., 2017). Previous evidence shows that missense mutations at *TP53* gene were more frequent in patients with *Schistosoma japonicum*-induced rectal cancer than in non-schistosomiasis rectal cancer, whereas *Schistosoma mansoni* targets p53 altering its expression and may cause somatic mutations in *BCL2* and *C-Myc* oncogenes inducing colorectal cancer (van Tong et al., 2017). In leishmaniasis ROS and RNS, produced as a host defence mechanism, may lead to DNA strand breaks and increase cancer risk. Mononuclear leukocytes from infected patients presented increase DNA damage compared to healthy subjects (Oliveira and Cecchini, 2000; Kocyigit et al., 2005; Almeida et al., 2013). These results suggest that pathogen-induced DNA damage play a key role in cellular transformation (van Tong et al., 2017).

### Cell cycle regulation in pathogen infection and cancer progression

Cell cycle dysregulation, *i.e.* constant cell proliferation and apoptosis inhibition, upon pathogen infection is a frequent process that promotes cancer onset (Machicado and Marcos, 2016). Function of p53, a key player in cell cycle progression, is altered during *Theileria* spp. and *Cryptosporidium parvum* infections possibly due to its cellular mislocalization or continuous degradation favouring cell malignant transformation (Hayashida et al., 2013; Benamrouz et al., 2014). Alterations in *APC* and *β-catenin* genes, involved in Wnt signalling pathway, are altered in *C. parvum* infection affecting actin cytoskeleton organization and other cellular processes that might result in neoplasia development (Benamrouz et al., 2014). This cell cycle dysregulation is also observed in viral infection through the production of oncogenic factors. For example, Human T-cell leukaemia virus type 1 synthesizes Tax, a viral replication protein, which is responsible for the activation of Wnt and NF-Kb pathways and the inhibition of DNA repair resulting in cell malignant transformation. (Yasunaga and Matsuoka, 2018). In leishmaniasis the parasite modulates host cell survival and death; *Leishmania* through released factors or surface proteins, such as the lipophosphoglycan, hijack the apoptosis machinery leading to its inhibition in infected macrophages (Ouaissi and Ouaissi, 2005), whereas elevated oxidative stress during infection leads to the death of other immune cell types (Almeida et al., 2013).

### Immune response evasion, a common survival strategy of pathogens and cancer cells

An immunological association between pathogen infection and cancer has been suggested (Ouaissi and Ouaissi, 2005; Silmon de Monerri and Kim, 2014; Machicado and Marcos, 2016; Schwing et al., 2019). Similar strategies are orchestrated by pathogens and tumour cells to evade host immune response enabling their survival and proliferation (Ouaissi and Ouaissi, 2005). *Leishmania* spp. immune-modulate host system activating Th2 response and reducing Th1 protective defence. In visceral leishmaniasis caused by *L. chagasi* this altered balance between Th1 and Th2 may cooperate to acute leukaemia development (de Vasconcelos et al., 2014). This immune environment favours infection progression avoiding pathogen killing and it increases susceptibility to tumour formation as well (Ouaissi and Ouaissi, 2005; Machicado and Marcos, 2016; Schwing et al., 2019). Moreover, previous evidence shows that *T. cruzi* and *Leishmania* spp. manage to kill immune cells and inhibit apoptosis of host cells. Alteration of apoptotic pathways is a strategy employed also by tumours to prevent their clearance by immune cells (Ouaissi and Ouaissi, 2005).

### Pathogen coinfections

Association between infectious diseases and cancer development become more complex when simultaneous pathogen infections occur, possibly cooperating to tumorigenesis by triggering cell proliferation, inflammation and inducing genomic/epigenomic alterations (van Tong et al., 2017; Yasunaga and Matsuoka, 2018). For example, *Strongiloides stercolaris*, the pathogen agent of a chronic gastrointestinal parasitic infection, promotes proliferation of human T-cell leukaemia virus type 1-infected cells increasing the risk to develop adult T-cell leukaemia/lymphoma (Gabet et al., 2000; Yasunaga and Matsuoka, 2018). *P. falciparum* and EBV are associated with BL, a frequent children tumour in tropical Africa. BL has been diagnosed in hyperendemic malaria regions and EBV was first discovered in BL tumour biopsies (Torgbor et al., 2014). *P. falciparum* interacts with B cells and impairs their function leading to an uncontrolled proliferation including EBV-infected B cells and on the other hand the parasite inhibits EBV-specific T cell immunity reactivating virus cycle and promoting chromosome aberrations. This environment may lead to the onset and expansion of malignant B-cells and consequent BL development (van Tong et al., 2017). BL has been also reported in a human immunodeficiency virus (HIV)-infected subject presenting leishmaniasis (Boutros et al., 2006; Schwing et al., 2019). HIV-mediated immunosuppression facilitates parasite replication and dissemination, whereas *Leishmania* interferes with monocytes and macrophages normal function increasing dNTP levels, essential for efficient HIV replication and thus favouring HIV progression (Mock et al., 2012; Zijlstra, 2014). Moreover, few cases of Kaposi sarcoma and *Leishmania* infection in HIV-positive patients have been reported, all these patients presented typical lesions of Kaposi sarcoma with high *Leishmania* parasitaemia (Kopterides et al., 2007). No association with malignant transformation has been reported in cases of coinfection of *Leishmania* and other pathogens, such as *P. falciparum* and *T. cruzi* (Martinez et al., 2018).

### DNA methylation, an epigenetic sign, linking infectious disease and cancer onset

DNA methylation is a fascinating landscape that needs to be further explored in infectious diseases. Few works focused their attention on DNA methylation alterations upon pathogen infection highlighting their importance during host cell colonization and suggesting an increased predisposition to develop cancer. These aberrations may result in transcriptional dysregulation affecting normal cell function and the surrounding environment (Silmon de Monerri and Kim, 2014; Robert McMaster et al., 2016). *H. pylori* infection induces changes in DNA methylation at the promoter regions of *THBS1*, *GATA-4* and *FOXD3*, associated with angiogenesis, cell differentiation and apoptosis, promoting gastric cancer development. Interestingly, some of these aberrations are long-lasting modifications that persist after eradication of the bacteria (Alvarez et al., 2013; Cheng et al., 2013; Silmon de Monerri and Kim, 2014). In EBV infection, activation of DNA methyltransferases and transcriptional regulation of *BIM*, an apoptosis inducer, and *CDH1*, a cell-cell adhesion protein, may also play a key role in gastric cancer onset (Paschos and Allday, 2010; Silmon de Monerri and Kim, 2014). Another correlation has been indicated between the presence of HBV in hepatocellular carcinoma and aberrant DNA methylation in host cells. In fact, a nuclear viral protein regulates the expression of DNMT1 and DNMT3A enzymes, relevant players in mechanisms that modulate the methylome (Paschos and Allday, 2010). Human papillomavirus (HPV) infection is considered an important risk factor for cervical cancer development. Two viral oncoproteins, E6 and E7, alter cellular DNA methylation of tumour suppressor genes, such as *CCNA1*, *CADM1* and *DAPK1*, through the formation of a complex with DNMT1 and probably with transcription factors (Woodman et al., 2007; Yanatatsaneejit et al., 2020; Singh et al., 2022). HPV-linked methylation aberrations are useful for prognosis and identification of cervical cancer subtypes (Yang et al., 2020). As mentioned previously, it is well known the involvement of DNA methylation alterations, despite their aetiology, in tumours. DNA methylation alterations may dysregulate gene expression and affect a cascade of events promoting tumorigenesis (Feinberg and Tycko, 2004) and they are also considered specific tumour signatures useful for prevention and diagnosis since they precede mutations (Kulis and Esteller, 2010). However, cancer development is the result of the accumulation of genetic and epigenetic alterations (Kulis and Esteller, 2010). **Table 1** summarizes the DNA methylation-related alterations detected in pathogen infection-associated cancer.

**Table 1:** DNA methylation-related alterations in pathogen infection-associated cancer.

	Affected genes/proteins	Function	Associated pathogen	Associated cancer	References
Genes with altered methylation	***THBS1*** (Thrombospondin 1)	Angiogenesis inhibitor	*Helicobacter pylori*	Gastric cancer	(Alvarez et al., 2013)
	***GATA-4*** *(GATA Binding Protein 4)*	Cellular specification and differentiation promoter	*Helicobacter pylori*	Gastric cancer	(Alvarez et al., 2013)
	***FOXD3*** (Forkhead Box D3)	Transcriptional regulator in several biological processes	*Helicobacter pylori*	Gastric cancer	(Cheng et al., 2013; Silmon de Monerri and Kim, 2014)
	***BIM*** (Bcl-2 Interacting Mediator Of Cell Death)	Apoptosis inducer	Epstein–Barr virus	Gastric cancer	(Paschos and Allday, 2010; Silmon de Monerri and Kim, 2014)
	***CDH1*** (E-cadherin 1)	Cell-cell adhesion protein	Epstein–Barr virus	Gastric cancer	(Paschos and Allday, 2010)
	***CCNA1*** (Cyclin-A1)	Cell cycle regulator	Human papillomavirus	Cervical cancer	(Woodman et al., 2007; Yanatatsaneejit et al., 2020; Singh et al., 2022)
	***CADM1*** (cell adhesion molecule 1)	Cell adhesion protein; cell junction organizer	Human papillomavirus	Cervical cancer	(Yanatatsaneejit et al., 2020; Singh et al., 2022)
	***DAPK1*** (death associated protein kinase 1)	Apoptosis inducer	Human papillomavirus	Cervical cancer	(Yanatatsaneejit et al., 2020; Singh et al., 2022)
Altered DNMTs (DNA Methyltransferases)	**DNMTs** expression and activation	DNA methylation inducer	Epstein–Barr virus	Gastric cancer	(Paschos and Allday, 2010; Silmon de Monerri and Kim, 2014)
	**DNMTs** expression	DNA methylation inducer	Hepatitis B virus	Hepatocellular carcinoma	(Paschos and Allday, 2010)

## May DNA methylation alterations in leishmaniasis trigger tumorigenesis?

Long-term *Leishmania* infection causes chronic inflammation favouring accumulation of epigenetic and genetic mutations that can promote cancer development. During acute and chronic infection, amastigotes ensure their survival within phagolysosomes developing semi-quiescent and dormant stages, *i.e.* reducing or shutting down expensive metabolic processes and maintaining a slow or very slow growth (Saunders et al., 2021). *Leishmania* chronic infection and cancer lead to a susceptible environment allowing the progression of both diseases (Al-Kamel, 2017; Schwing et al., 2019). It is known that many cancers arise from infection sites (Al-Kamel, 2017). The pathophysiologic contribution of *Leishmania* spp. in cancer onset is not well elucidated but many patients with prior *Leishmania* spp. infection developed tumours (Al-Kamel, 2017). To note, it is believed that approximately 120 million people have a chronic infection (Saunders et al., 2021), thus monitoring infection parameters achieve great relevance to prevent and early diagnose cancer in endemic regions. The tumour cases reported in patients with leishmaniasis included basal and squamous cell carcinoma, lymphoma, leukaemia, hemangiosarcoma and hepatocellular carcinoma, summarized in Al-Kamel (Al-Kamel, 2017).

**Figure 3:**
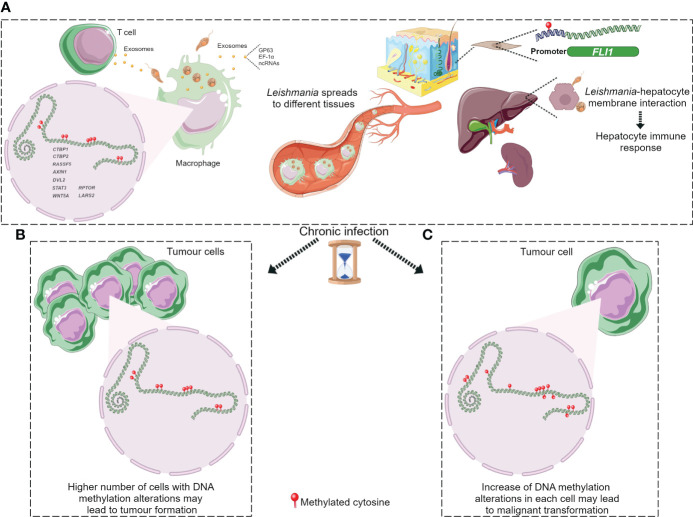
DNA methylation alterations upon *Leishmania* infection possibly leading to tumorigenesis. **(A)** *Leishmania* molecules, possibly delivered by exosomes, induce DNA methylation changes in macrophages and maybe in T cells. Parasite dissemination to different tissues may induce methylome alterations in other cell types, such as fibroblasts and hepatocytes, through *Leishmania*/host cell interaction. Cancer development may arise through different ways during chronic leishmaniasis: **(B)** increased number of cells with DNA methylation alterations; **(C)** additional methylome alterations in target cells. Figure created using Servier Medical Art images, licensed under a Creative Commons Attribution 3.0 Unported License; https://smart.servier.com/.

DNA methylation aberrations induced by *Leishmania* spp. infection may be a possible factor that trigger cell malignant transformation since DNA methylation alterations are frequent early events in cancer. It has been reported that a macrophage cell line infected with *L. donovani* displays DNA methylation changes in loci linked to pathways frequently altered in cancer (Marr et al., 2014). The affected genes were *CTBP1*, *CTBP2*, *RASSF5*, *AXIN1*, *DVL2*, *STAT3* and *WNT5A* (**Table 2**) (Marr et al., 2014). Some of these gene-related pathways have been above described in pathogen infection-associated cancer. *AXIN1*, *DVL2* and *WNT5A* are involved in Wnt signalling that regulates several processes such as proliferation, differentiation and adhesion. Wnt5A has been related to cancer-associated inflammation through macrophage recruitment. CTBPs corepressors are involved in cell cycle and *WNT* gene family regulation (Stankiewicz et al., 2014; Blevins et al., 2017). As aforementioned, Wnt pathway abnormal activation and mutations or mislocalization of its members are frequently observed in infectious diseases and are considered possible tumour promoters (Benamrouz et al., 2014; Mazzoni and Fearon, 2014; Asem et al., 2016; Yasunaga and Matsuoka, 2018; Mei et al., 2020). RASSF5 may be implicated in p53 activity regulation, a key cell cycle guardian frequently altered in infectious diseases leading to cancer onset (Hayashida et al., 2013; Benamrouz et al., 2014; Li et al., 2018). DNA methylation aberrations may explain the altered expression of these genes and thus signalling pathways dysregulation. In fact, these altered loci showed a significant differential methylation with a range from 3% to 9% between infected and non-infected cells (Marr et al., 2014). Notably, although these alterations are located in the gene body and not in regulatory regions, they may also modify gene expression. Long-term consequences of chronic infection in the epigenetic modulation may lead to further methylome alterations in the target cells such as T cells, hepatocytes and fibroblasts, and an increased number of infected cells than in previous phases of infection that may induce aberrations in neighbour cells, probably contributing to the occurrence of malignancy (**Figure 3**). Additional DNA methylation aberrations in target cells may be caused by *Leishmania* released molecules delivered by extracellular vesicles, whereas a higher number of infected macrophages may enhance parasite dissemination to visceral organs, resulting in amastigotes and/or exosome release towards other target cells. In agreement with this statement, few works reported cancer-specific methylation alterations several years before mature B-cell neoplasm (MBCN) diagnosis (Wong Doo et al., 2016; Georgiadis et al., 2017; Loi et al., 2019). In fact, in a prospective MBCN cohort analysis Loi et al. found a hypermethylation event at a CGI associated with *SHANK1* gene (differential methylation of 3%) in blood samples collected around 10 years prior to diagnosis (Loi et al., 2019). This evidence supports the hypothesis that weak DNA methylation changes observed upon *Leishmania* spp. infection may enhance during long-term infection predisposing tumorigenesis (**Figure 3**). To further support the possible association between *Leishmania* spp. infection and cancer onset susceptibility, DNA methylation alterations at *FLI1* promoter region, a gene dysregulated in melanoma, have been detected in macrophages infected with *L. braziliensis in vitro* and in fibroblasts from cutaneous lesion biopsies as well (Almeida et al., 2017; Ramani et al., 2017). To note, it is believed that epigenetic modulation, including DNA methylation, has a main role in fibroblasts transformation into cancer-associated fibroblasts (CAFs) and this process can be mediated by exosomes, a frequent vehicle used in parasite/host communication. CAFs are an essential component of tumour microenvironment and they can also infiltrate and metastasize together with tumour-specific cells (Ping et al., 2021). Cancer onset in leishmaniasis wounds could arise even several years after apparent healing reinforcing the relevance of chronic infections and the persistence of parasite-associated alterations in host cells. Some cases of basal cell carcinoma in patients with *Leishmania* infection have been reported. Therefore, cutaneous leishmaniasis can be considered a predisposing factor for skin malignancies (Suster and Ronnen, 1988; Morsy et al., 1992; Yavuzer et al., 2001; Mangoud et al., 2005; Asilian et al., 2012; Morsy, 2013; Schwing et al., 2019). Another type of tumour reported in patients with leishmaniasis is the T-cell lymphoma that may arise in consequence of chronic antigenic stimulation and immunosuppression due to leishmaniasis (Al-Kamel, 2017). Interestingly, *Leishmania* vesicles target T cells altering their function (Silverman et al., 2010b; Coakley et al., 2015). In 2003, a case report of a patient with hepatocellular carcinoma with pre-existing visceral leishmaniasis and chronic hepatitis C has been published (Precone et al., 2003; Kopterides et al., 2007). As aforementioned, it has been reported *Leishmania*/hepatocyte membrane interaction and gene expression alterations in these target cells (Rodrigues et al., 2019). Therefore, it is reasonable to wonder whether this interplay induces host DNA methylation changes leading to gene dysregulation, cell dysfunction and probably cell malignant transformation in long-term infections. Further studies are needed to elucidate a possible association between visceral leishmaniasis and cancer onset.

**Table 2:** Cancer pathway’s genes, associated with altered CpG sites methylation upon infection with *L. donovani*.

Gene name	Function	Associated cancers	References
***CTBP1*** (C-terminal binding protein 1)	Transcription corepressor	Melanoma, leukaemia, breast and colon cancer, among others	(Blevins et al., 2017)
***CTBP2*** (C-terminal binding protein 2)	Transcription corepressor	Breast, colon and lung cancer, among others	(Stankiewicz et al., 2014)
***RASSF5*** (Ras Association Domain Family Member 5)	Tumour suppressor	Colorectal cancer, hepatocellular carcinoma, osteosarcoma, among others	(Li et al., 2018)
***AXIN1***	Signalling regulator in Wnt pathway	Hepatocellular carcinoma; colon and gastric cancer, among others	(Mazzoni and Fearon, 2014)
***DVL2*** (Dishevelled Segment Polarity Protein 2)	Key factor in signal transduction (Wnt pathway)	Hepatocellular carcinoma; breast cancer	(Mei et al., 2020)
***STAT3*** (signal transducer and activator of transcription 3)	Transcription activator	Colorectal cancer, hepatocellular carcinoma, breast cancer, T cell/B cell lymphoma, non-small cell lung cancer, among others	(Yu et al., 2014)
***WNT5A*** (Wingless-Type MMTV Integration Site Family, Member 5A)	Secreted signalling protein (Wnt pathway)	Melanoma, chronic lymphocytic leukaemia, thyroid, colorectal, gastric and ovarian cancer, among others	(Asem et al., 2016)

DNA methylation alterations induced by *Leishmania* spp. may result in an immune response dysregulation that leads to a non-correct surveillance and enables eventual malignant cells at the leishmanial lesion to elude immune detection (Al-Kamel, 2017). *Leishmania* infection activates mTOR pathway triggering cell proliferation and altering the balance of M1/M2 macrophages polarization, a biological process regulated by DNA methylation modifications and other mechanisms (Li et al., 2020). The increased M2 macrophage polarization represents an appropriate niche for *Leishmania* survival and on another hand it has a key role in angiogenesis, tumour formation and progression (Kumar et al., 2018; Yao et al., 2019; Zou et al., 2020). However, it is not elucidated how mTOR signalling is activated in leishmaniasis (Kumar et al., 2018). Marr et al. reported DNA methylation alterations in CpG sites associated with *LARS2* and *RPTOR* genes, involved in the mTOR pathway (Marr et al., 2014). These authors also reported methylation alterations at *IL-10*, an M2 macrophage marker, frequently altered in inflammation processes and secreted by many tumour types to suppress T cells response (Ouaissi and Ouaissi, 2005; Marr et al., 2014). **Figure 3** depicts the hypothesis described in this section regarding cancer onset in patients with leishmaniasis.

## Discussion

In this review we propose a possible mechanism for cancer onset during leishmaniasis. The protozoan *Leishmania* manipulates the epigenome, including DNA methylation, of host cells to enable its survival and replication (Robert McMaster et al., 2016). Changes in the DNA methylation profile may lead to gene expression dysregulation and cell dysfunction. Several DNA methylation modifications due to *Leishmania* infection are associated with genes involved in cancer pathways such as Wnt signalling and cell cycle regulation, and in immune mechanisms including cytokines signalling and Natural killer cells-mediated cytotoxicity (Marr et al., 2014). Case reports describe cancer onset in patients with long term infection suggesting the persistence or even worsening of the cell and tissue alterations caused by *Leishmania* (Kopterides et al., 2007; Schwing et al., 2019). DNA methylation alterations are also early events during tumorigenesis and few events of DNA methylation changes can be found even 10 years before cancer onset (Wong Doo et al., 2016; Georgiadis et al., 2017; Loi et al., 2019). Considering these characteristics, it is plausible that the association between parasitic infection and cancer may reside in these epigenetic changes. Long-term *Leishmania* infection may lead to a high number of altered cells and further increase DNA methylation aberrations in host cells enhancing patient susceptibility to cancer onset. An increased number of infected macrophages may allow parasite dissemination and induce methylome alterations in neighbour cells. These alterations are possibly induced by *Leishmania* molecules delivered by exosomes to different cell types including T cells, fibroblasts and hepatocytes resulting in cell dysfunction. Further studies are necessary to explore parasite/host cells interplay, including non-immune cells, to elucidate the consequences of DNA methylation changes during infection. Since a correlation between host DNA methylation alterations and cancer risk has been reported in other pathogen diseases such as *H. pylori*, EBV, HBV and HPV infection, it can be expected a similar mechanism in leishmaniasis. The comprehension of these molecular aberrations and the association between leishmaniasis and cancer could be useful to monitor infected patients in constant follow up and thus the progression of both pathologies.

## Data availability statement

The original contributions presented in the study are included in the article/supplementary material. Further inquiries can be directed to the corresponding author.

## Author contributions

Conceptualization: AFV-B and PZ; literature search, AFV-B; writing original draft preparation: AFV-B; writing, review and editing: AFV-B, EL, and PZ; visualization: AFV-B; funding acquisition: AFV-B. All authors contributed to the article and approved the submitted version.

## Funding

This study has been funded by a Research Grant [2021] from the European Society of Clinical Microbiology and Infectious Diseases (ESCMID) to AV-B.

## Conflict of interest

The authors declare that the research was conducted in the absence of any commercial or financial relationships that could be construed as a potential conflict of interest.

## Publisher’s note

All claims expressed in this article are solely those of the authors and do not necessarily represent those of their affiliated organizations, or those of the publisher, the editors and the reviewers. Any product that may be evaluated in this article, or claim that may be made by its manufacturer, is not guaranteed or endorsed by the publisher.
